# Mechanisms of Lushi Runzao decoction in Treating Sjögren’s syndrome by remodeling gut flora to regulate bile acid and short-chain fatty acid metabolism

**DOI:** 10.3389/fphar.2025.1505642

**Published:** 2025-06-26

**Authors:** Fengtao Pang, Quan Jiang, Xiaopo Tang, Kesong Li

**Affiliations:** Department of Rheumatology, Guang’anmen Hospital, Chinese Academy of Chinese Medical Sciences, Beijing, China

**Keywords:** Sjögren’s syndrome, Lushi Runzao decoction, gut flora, metabolomics, bile acid, short-chain fatty acid

## Abstract

**Objective:**

To explore the mechanisms of Lushi Runzao decoction (LSRZT) in Treating Sjögren’s Syndrome (SS) through gut flora, short-chain fatty acid (SCFAs) and metabolomics.

**Methods:**

Non Obese Diabetes (NOD) /LtJ mice were used as the model group, LSRZT and hydroxychloroquine (HCQ) were administered to the treatment group. Fecal samples were collected 4 weeks after the intervention. The microbiota, SCFAs and metabolites were analyzed using 16S ribosomal Ribonucleic Acid, Gas chromatography mass spectrometry analysis and Ultra High Performance Liquid Chromatography-Quadrupole Time-of-Flight Mass Spectrometry.

**Results:**

LSRZT had obvious anti-inflammatory effect and can effectively improve the functional injury of submandibular gland; It improves the imbalance of gut microbiota and the related metabolic levels of SCFAs and bile acids, and regulates the levels of inflammatory factors and the contents of bile acids and caproic acid by regulating the abundance of Erysipelatoclostridiaceae, Gammaproteobacteria and Ruminococcaceae.

**Conclusion:**

LSRZT can reduce the level of inflammation and improve the imbalance of gut microbiota and metabolism. It provides a scientific basis for the clinical treatment of SS by LSRZT.

## 1 Introduction

Sjogren’s syndrome (SS) is a systemic autoimmune disease characterized by salivary gland dysfunction, lymphocyte infiltration, and autoantibodies. The primary symptoms include dryness of the mouth and eyes, fatigue, and joint pain (Nocturne, 2019). SS is a global disease affecting over 90% of women, with onset typically occurring in middle-aged and elderly individuals aged 40–60 (Ramos-Casals et al., 2012). The onset is hidden and lacks specificity and can involve other systems such as the respiratory system, digestive system, blood system, muscles, and joints, resulting in multisystem and multi-organ damage (Selmi and Gershwin, 2017). However, its pathogenesis remains unclear. Several factors contribute to SS, including heredity, infections, and environmental influences (Jonsson, 2022). The primary cause is tissue damage and inflammation resulting from an abnormal immune system response to autoantigens, characterized by excessive proliferation of B cells and a reduction in T cells. Most treatments rely on glandular secretion drugs, immunosuppressants, and glucocorticoids to alleviate the patient’s symptoms. The drug primarily used in the clinic is hydroxychloroquine (HCQ) (Kida et al., 2024; Nirk et al., 2020). However, these approaches do not address the underlying causes, may lead to certain toxic and adverse effects, and are prone to recurrence (Gottenberg et al., 2014; Dörner et al., 2019; Baer et al., 2021; Felten and Gottenberg, 2023). Therefore, there is a pressing need for the development of safer and more effective drugs for the clinical treatment of SS.

Due to its characteristics of multi-targeting, multi-molecules, and safety and effectiveness, Traditional Chinese Medicine (TCM) has attracted global attention. TCM provides distinct advantages in alleviating SS progression and regulating the physical and mental burden (Wang et al., 2021). Lushi Runzao decoction (LSRZT) is created by Professor Lu Zhizheng, a master of TCM, based on his decades of experience in medical practice and pharmacology, and grounded in TCM theory. It has multiple therapeutic effects, including benefiting qi and nourishing yin, clearing heat and moistening dryness, generating fluids and relieving thirst, promoting blood circulation and resolving stasis, as well as transforming phlegm and unblocking channels. It is primarily used to treat SS and can effectively improve patients’ symptoms and quality of life Clinical study has proved that LSRZT can significantly alleviate the symptoms of SS, including dry mouth, dry eyes, and fatigue. Pharmacological study indicates that LSRZT can alleviate damage to submandibular gland tissue and reduce inflammation levels (Fengtao et al., 2023). Although the clinical efficacy of LSRZT has been established, its mechanism of action remains unclear.

Gut flora plays a crucial role in innate and acquired immunity in humans, its imbalance and abnormality is closely related to the occurrence and development of autoimmune diseases, which can disrupt the intestinal mucosal barrier, leading to intestinal mucosal immune dysfunction and the release of a large number of Interleukin (IL)-1, IL-17, and Tumor Necrosis Factor Superfamily Member (TNFSF) 13, thereby inducing chronic inflammation (Turroni et al., 2008; Zoetendal et al., 2006; Bäckhed et al., 2005). A study indicated that the occurrence of SS is closely linked to an imbalance in gut flora, chronic inflammation resulting from this imbalance is a significant immunopathological mechanism contributing to SS (Clemente et al., 2018). Another study on gut flora related to SS indicated that the diversity of gut flora in SS patients decreased significantly and the number of probiotics also decreased significantly compared to that in healthy individuals, the diversity of gut flora is negatively correlated with SS severity (de Paiva et al., 2016). The study combined with the clinical severity index of SS patients indicated that the exacerbation of intestinal dysbiosis might be positively correlated with the worsening of its systemic manifestations and laboratory indicators (Mandl et al., 2017). Therefore, gut flora is an important target for treating SS. 16S ribosomal Ribonucleic Acid (16S rRNA) sequencing technology is a high-throughput sequencing method that can accurately identify the species and abundance of gut microbiota. Short-Chain Fatty Acids (SCFAs), primarily produced by the metabolism of gut flora, include substances such as acetate, propionate, and butyrate, which can regulate host immunity, metabolism, and inflammatory responses. SCFAs are closely related to SS. studies showed that SCFAs inhibit the activation of autoreactive B cells by upregulating Tregs (CD4^+^CD25^+^Foxp3^+^) (Zhang et al., 2024), reducing the production of anti-SSA/SSB antibodies, lowering serum levels of IL-17 and IFN-γ in SS patients, and suppressing glandular destruction driven by Th1/Th17 (Szymula et al., 2014). Metabonomics technology reveals changes in the comprehensive metabolic system by thoroughly analyzing the entire metabolic spectrum of various organisms, it plays an important role in analyzing disease metabolic pathways (Trifonova et al., 2023).

In this study, we analyzed the main targets and pathways of LSRZT in the treatment of SS through network pharmacology. We used Ultra Performance Liquid Chromatography-Mass Spectrometry (UPLC-MS)/MS to examine the main components of LSRZT and validated its effects on reducing inflammation and protecting salivary gland tissue function in SS using methods such as Enzyme-linked immunosorbent assay (ELISA) and flow cytometry. Additionally, we explored the mechanism of LSRZT in treating SS by regulating gut microbiota abundance, SCFAs levels, and metabolite levels by 16S rRNA, Gas Chromatography-MS, and metabolomics.

## 2 Materials and methods

### 2.1 Preparation of LSRZT

LSRZT comprises the following drugs: *Pseudostellaria heterophylla (Miq.) Pax ex Pax et Hoffm.* [Caryophyllaceae; Radix Pseudostellariae] (No.Y11-007-0) 15 g, *Dioscorea opposita Thunb.* [Dioscoreaceae; Rhizoma Dioscoreae] (No.Y11-034-0) 15 g, *Ophiopogon japonicus (L. f.) Ker Gawl.* [Asparagaceae; Radix Ophiopogonis] (No.Y11-022-2) 15 g, *Glehnia littoralis F. Schmidt ex Miq.* [Apiaceae; Radix Glehniae] (No.Y11-005-0) 15 g, *Salvia miltiorrhiza Bunge* [Lamiaceae; Radix et Rhizoma Salviae] (No.Y11-009-0) 10 g, *Paeonia veitchii Lynch* [Paeoniaceae; Radix Paeoniae Rubra] (No.Y11-057-0) 10 g, *Citrus medica L.* var. *sarcodactylis Swingle* [Rutaceae; Fructus Citri Sarcodactylis] (No.Y12-109-1) 10 g, *Atractylodes macrocephala Koidz.* [Asteraceae; Rhizoma Atractylodis Macrocephalae] (No.Y11-024-0) 10g, *Pueraria montana (Lour.) Merr. var. lobata (Willd.) Sanjappa & Pradeep* [Fabaceae; Radix Puerariae] (No.Y11-071-0) 10 g, *Zaocys dhumnades (Cantor)* [Colubridae; Zaocys] (No.Y18-070-0) 10 g, *Dendrobium nobile Lindl.* [Orchidaceae; Caulis Dendrobii] (No.Y13-017-0) 10 g, *Rehmannia glutinosa (Gaertn.) DC.* [Orobanchaceae; Radix Rehmanniae] (No.Y11-018-0) 10 g, *Gentiana macrophylla Pall.* [Gentianaceae; Radix Gentianae Macrophyllae] (No.Y11-064-0) 10 g. These drugs are provided by the Pharmacy Department of Guang’anmen Hospital, Chinese Academy of Chinese Medical Sciences. All the drugs are first soaked in water at a volume 10 times that of the materials for 30 min, and then boiled for 30 min. The mixture is filtered through four layers of gauze, and the filtrate is boiled again with water at a volume four times that of the filtrate. The extract is concentrated to 200 mL using a rotary evaporator and stored at 4°C for future use. LSRZT was prepared by the Preparation Center of Guang’anmen Hospital, Chinese Academy of Chinese Medical Sciences.

### 2.2 Component analysis of LSRZT

Methanol was added to the LSRZT water extract, and the mixture was centrifuged to obtain the supernatant for UPLC-MS/MS analysis. A Vanquish Flex UPLC chromatograph (Thermo Fisher Scientific, Inc., Waltham, MA, United States) equipped with an ACQUITY UPLC HSS T3 column (2.1 mm (inner diameter) ×100 mm (length), 1.7 μm (particle dimension), Waters Corp., MA, United States) was used for separation. The mobile phase was consisted of water (0.1% formic acid, phase A) and acetonitrile (phase B) with a flow rate of 0.3 mL/min and the column temperature was 40°C. The injection volume was 6.0 µL. The multi-step liner elution gradient program was set as follows: 0–1 min, 98%–98% A; 1–14 min, 98%–70% A; 14–25 min, 70%–0% A; 25–28 min, 0%–0% A; 28–28.1 min, 0%–98% A; 28.1–30 min, 98%–98% A. The MS data was processed by Progenesis QI 3.0 (Waters Corp., MA, United States) with the steps of raw data introduction, peak extraction and adduct deconvolution. The MS data was collected by a hybrid quadrupole orbitrap mass spectrometer (Q Exactive, Thermo Fisher Scientific, Inc., Waltham, MA, United States) equipped with a HESI-II spray probe. The parameters were set as follows: positive ion source voltage 3.7 kV and negative ion source voltage 3.5 kV, heated capillary temperature 320°C, sheath gas pressure 30 psi, auxiliary gas pressure 10 psi, desolvation temperature 300°C. Both the sheath gas and the auxiliary gas were nitrogen. The collision gas was also nitrogen with a pressure of 1.5 mTorr. The data was acquired in “Full scan/dd-MS^2^” mode. The parameters of the full scan were set as follows: resolution 7,000, auto gain control target 1 × 10^6^, maximum isolation time 50 m. The dd-MS^2^ data was collected with the parameters of resolution 17,500, auto gain control target 1 × 10^5^, maximum isolation time 50 m, loop count of top 10 peaks, isolation window of m/z 2, collision energy 10 V, 30 V, 60 V and intensity threshold 1 × 10^5^.

### 2.3 Network pharmacology analysis

Active ingredients of LSRZT were screened from the TCMSP database using dual thresholds of oral bioavailability (OB ≥ 30%) and drug-likeness (DL ≥ 0.18). Corresponding targets were mapped to human genes via UniProt (species: *Homo sapiens*) and cross-referenced with Sjögren’s syndrome (SS)-associated targets retrieved from GeneCards (Relevance Score >10). A total of 76 overlapping targets were identified through Venn analysis and validated using the STRING database (interaction confidence ≥0.9; species: *Homo sapiens*). Target prioritization integrated network topology parameters (degree and betweenness centrality calculated via Cytoscape 3.7.2) and functional enrichment (DAVID-based KEGG pathway analysis, FDR <0.05), with composite scores weighted toward SS-relevant pathways. The resultant component-target-pathway network highlighted multi-target components mechanistically linked to immunomodulation and glandular function restoration in SS.

### 2.4 Animal grouping and drug administration

Twelve SPF-grade female BALB/c mice (8 weeks old) and thirty-six SPF-grade female Non Obese Diabetes (NOD)/LtJ mice (8 weeks old) were procured from Beijing Huafukang Biotechnology Co. Ltd. (license No. SCXK (Beijing) 2019–0008. Throughout the experimental phase, the mice were accommodated at the Animal Experimentation Center of Guang’anmen Hospital, China Academy of Chinese Medical Sciences, with 6 mice housed per cage. Deionized water for animals was provided in 50 mL plastic bottles for consumption, and the water was refreshed daily. The control group consisted of 12 Balb/c mice. NOD/LtJ mice were randomly divided into three groups based on body weight: model, LSRZT, and HCQ groups, with 12 mice in each group. LSRZT or HCQ was continuously administered to each group by gavage from 9 weeks of age. The model group was administered an equal volume of deionized water once daily for 4 weeks until the end of the experiment. HCQ was produced by the Shanghai Pharmaceutical Company under batch number H19990263. The dosage conversion formula for LSRZT is defined as Dm = Dh/W *F (Ren et al., 2019; Liu et al., 2025), where Dm represents the dose given to mice, Dh is the clinical dose for human, W represents the average adult weight (assumed to be 60 kg), and F is the conversion factor between rat and human doses, set at 9.01. Therefore, the standard oral dose of LSRZT for mice (20 g) is 450 mg/(kg·day), and the dose of HCQ is 1.2 mg/(kg·day). After continuous gavage for 4 weeks, the mice received an intraperitoneal injection of 10% chlorohydrate anesthetic (0.3 mL/100 g body weight). Blood was collected from the abdominal aorta, and then we separated the submandibular gland, spleens, and feces from the intestines, which were stored at −80°C. This study was conducted with ethical clearance by the Medical Ethics Committee of Guang’anmen Hospital, China Academy of Chinese Medical Sciences, on 4 June 2024 (Approval No: 2024–109-KY).

### 2.5 Measurement of water consumption and salivary flow rate

During the experimental period, water intake was measured weekly at fixed time intervals. Fresh drinking water was provided to mice, and the daily water consumption per group was calculated as the difference between the pre-measured water volume supplied the previous day (M1) and the remaining water volume on the measurement day (M2). For each group containing twelve mice, the daily water intake per mouse was determined as (M1-M2)/12. The average daily water consumption per mouse was recorded throughout the treatment period.

Salivary flow rate was assessed weekly using the cotton ball saturation method. Mice were anesthetized via inhalation of 1%–4% isoflurane, and anesthesia depth was confirmed by monitoring respiratory patterns, muscle tone, and corneal reflexes. Pre-weighed sterile cotton balls (M1) were positioned at the orifice of the submandibular gland ducts. After 15 min, the cotton balls were retrieved and reweighed (M2). Saliva secretion per mouse was quantified as M1-M2. All measurements were conducted using an electronic analytical balance to ensure precision.

### 2.6 Determination of submandibular gland and spleen index

Following the intervention period, mice were fasted for 12 h with *ad libitum* water access prior to body weight measurement. Animals were subsequently euthanized under anesthesia, and bilateral submandibular glands and spleens were surgically harvested. Organ indices were calculated as follows: Submandibular gland index = Total bilateral submandibular gland mass (mg)/Body weight (g); Spleen index = Spleen mass (mg)/Body weight (g). All excised organs were promptly weighed using a precision electronic balance to ensure measurement accuracy. Indices were expressed as milligram of tissue mass per gram of body weight (mg/g) to standardize comparisons across experimental groups.

### 2.7 Flow cytometry analysis

Spleens were immediately immersed in PBS post-excision for single-cell suspension preparation. Splenic single-cell suspensions were generated via mechanical dissociation followed by erythrocyte lysis. Aliquots containing 10^6^ cells per group were stained with fluorochrome-conjugated antibodies against CD3, CD4, CD25, CD279 (PD-1), and CXCR5, followed by incubation at 4°C for 30 min. Post-staining, cells were washed with 1 mL cell staining buffer and centrifuged at 300 *g* for 5 min. Supernatants were discarded, and pellets were resuspended in 100 μL staining buffer. Fixation was performed using 1 mL fixation buffer with vortex mixing, followed by 30 min incubation at 4°C. After fixation, cells underwent two permeabilization cycles with 2 mL permeabilization buffer (vortexed, centrifuged, and supernatant discarded). Cell pellets were resuspended in 100 μL permeabilization buffer and intracellularly stained with Foxp3 antibody for 30 min at room temperature. Following two additional permeabilization washes, cells were resuspended in 200 μL staining buffer and analyzed using a flow cytometer with appropriate compensation controls and gating strategies.

### 2.8 Histopathological analysis

The salivary gland of each mouse were collected for histopathological analysis and stained with hematoxylin and eosin (HE). Three experienced pathologists were asked to evaluate the Cutzler method. Each parameter was scored on a scale from 0 to 4, with higher scores indicating greater severity of salivary gland tissue injury. To determine the final severity score for each part of the salivary gland in mice, calculate the average score for each parameter and then sum these averages.

### 2.9 Serum levels of TNFSF13, IL-17, anti-SSA, and anti-M3R were measured using ELISA

The serum levels of TNFSF13, IL-17, anti-SSA and anti-M3R in mice were measured using a bispecific antibody sandwich ELISA. A total of 50 μL of standards at various gradient concentrations, along with serum samples diluted fivefold, were added to plates pre-coated with TNFSF13, IL-17, anti-SSA and anti-M3R antibodies. Subsequently, 100 μL of horseradish peroxidase-labeled detection antibody was added to each well and incubated at 37°C for 1 h, followed by five washes. Next, 50 μL of chromogenic substrates A and B were added to each well and protected from light at 37°C. When a distinct blue gradient appeared in the standard wells, a stop solution was added, and the optical density value was immediately measured at a wavelength of 450 nm. The concentrations of TNFSF13, IL-17, anti-SSA and anti-M3R were positively correlated with the optical density value, and the amounts of TNFSF13, IL-17, anti-SSA and anti-M3R in the samples were calculated using the standard curve equation and the dilution factor.

### 2.10 Determination of SCFAs in feces

Acetic acid, propionic acid, isobutyric acid, butyric acid, isovaleric acid, valeric acid,4-methylvaleric acid and caproic acid were all obtained from Sigma-Aldrich (Shanghai, China). Feces samples were homogenated for 1 min with 500 μL of water and 100 mg of glass beads, and then centrifuged at 4°C for 10 min at 12,000 rpm. 200 μL supernatant was extracted with 100 μL of 15% phosphoric acid and 20 μL of 375 μg/mL 4-methylvaleric acid solution as Internal Standard and 280 μL ether. Subsequently, the samples were centrifuged at 4°C for 10 min at 12,000 rpm after vortexing for 1 min and the supernatant was transferred into the vial prior to Gas chromatography mass spectrometry (GC-MS) analysis.

### 2.11 16S rRNA sequencing of fecal bacterial

Fecal samples were snap-frozen and stored at −80°C after collection. Bacterial DNA was isolated from the feces of mice using a DNeasy Power Soil kit (Qiagen, Hilden, Germany), following the manufacturer’s instructions. DNA concentration and integrity were measured using a NanoDrop 2000 spectrophotometer (Thermo Fisher Scientific, Waltham, MA, United States) and agarose gel electrophoresis, respectively. PCR amplification of the V3-V4 hypervariable regions of the bacterial 16S rRNA gene was conducted in a 25 μL reaction using universal primer pairs (343F: 5′-TACGGRAGGCAGCAG-3′, 798R: 5′-AGG​GTA​TCT​AAT​CCT-3′). The amplicon quality was visualized by gel electrophoresis. PCR products were purified using Agencourt AMPure XP beads (Beckman Coulter Co., United States) and quantified using a Qubit dsDNA Assay Kit. Sequencing was performed using an Illumina NovaSeq6000 with two paired-end read cycles of 250 bp each. Paired-end reads were preprocessed using Cutadapt software to identify and remove adapter sequences. 16S rRNA gene sequencing data were processed using QIIME 2 (V2020.11). Raw paired-end reads were demultiplexed, trimmed (--p-trim-left: 20 bp), and quality-filtered (--p-trunc-len: 250/240) using DADA2. Amplicon sequence variants (ASVs) were generated and taxonomically classified against the SILVA 138.1 database with a confidence threshold of 0.7. Alpha diversity (Shannon, Faith’s PD) and beta diversity (weighted UniFrac) metrics were calculated on rarefied data (10,000 reads/sample). Linear discriminant analysis Effect Size (LEfSe) was performed to calculate taxon abundance and to determine the differences among groups (linear discriminant analysis score (LDA) >3 and *P* < 0.05 were considered significant).

### 2.12 Metabolomics study on feces

32 fecal and 3 quality control (QC) samples were thawed at approximately 27°C and 50 mg of the sample was added to a 1.5 mL Eppendorf tube with 100 μL of L-2-chlorophenylalanine (0.3 mg/mL) dissolved in methanol as an internal standard for Ultra High Performance Liquid Chromatography - Quadrupole Time-of-Flight Mass Spectrometry (UHPLC-Q-TOF/MS). The tube was vortexed for 10 s. Subsequently, 100 μL of an ice-cold mixture of methanol and acetonitrile (2/1, vol/vol) was added, and the mixtures were vortexed for 1 min, and the whole samples were then extracted by ultrasonication for 10 min in an ice-water bath and stored at −20°C for 30 min. The extract was centrifuged at 4°C (13,000 × g) for 10 min. Next, 100 μL of supernatant was dried in a glass vial using a freeze concentration centrifugal dryer, and 100 μL mixture of methanol and water (1:4, v/v) was added to each sample. The samples were vortexed for 30 s, extracted by ultrasonication for 3 min in an ice-water bath, and then placed at −20°C for 2 h. Subsequently, the samples were centrifuged at 4°C (13,000 rpm) for 10 min. The supernatants (100 μL) from each tube were collected using crystal syringes, filtered through 0.22 μm microfilters, and transferred to LC vials. The vials were stored at −80°C until UHPLC-Q-TOF/MS analysis. QC samples were prepared by mixing aliquots of all samples to create a pooled sample. A Dionex Ultimate 3000 RS UHPLC system fitted with a Q-Exactive quadrupole-Orbitrap mass spectrometer equipped with a heated electrospray ionization (ESI) source (Thermo Fisher Scientific, Waltham, MA, United States) was used to analyze the metabolic profiles in both ESI positive and negative ion modes. An ACQUITY UHPLC HSS T3 column (1.8 μm, 2.1 × 100 mm) was employed in both positive and negative modes. The binary gradient elution system consisted of (A) water (containing 0.1% formic acid, v/v) and (B) acetonitrile (containing 0.1% formic acid, v/v), and separation was achieved using the following gradient: 0.01 min, 5% B; 2 min, 5% B; 4 min, 30% B; 8 min, 50% B; 10 min, 80% B; 14 min, 100% B; 15 min, 100% B; 15 min, 5% B; and 18 min, 5% B. The flow rate was 0.35 mL/min, and the column temperature was 45°C. All the samples were kept at 4°C during the analysis. The injection volume was 5 μL. The mass range was 100–1,000. The resolution was set to 70,000 for the full MS scans and 17,500 for the HCD MS/MS scans. The collision energies were set to 10, 20, and 40 eV, respectively. The mass spectrometer was operated as follows: spray voltage, 3,800 V (+) and 3,200 V (−); sheath gas flow rate, 40 arbitrary units; auxiliary gas flow rate, 8 arbitrary units; capillary temperature, 320°C; probe heater temperature, 350°C; S-lens RF level, 50. QC samples were injected at regular intervals (every 10 samples) throughout the analytical run to provide a dataset for assessing repeatability.

The original data were processed using the Progenesis QI V2.3 software (Nonlinear Dynamics, Newcastle, United Kingdom). Metabolite identification was based on the precise mass-to-charge ratio (m/z), secondary fragments, and isotopic distribution using the Human Metabolome Database (HMDB), Lipid Maps (V2.3), and Metlin databases for qualitative analysis. A data matrix was created by combining the positive and negative ion data. The matrix was imported into R to conduct partial least squares discriminant analysis (PLS-DA) to examine the overall distribution among the samples and the stability of the entire analysis process, taking VIP >1 as the standard of difference. To prevent overfitting, 7-fold cross-validation and 200 Response Permutation Testing were used to evaluate the quality of the model. Two-tailed Student’s t-test and one-way analysis of variance (ANOVA) were used to verify whether the differences in metabolites between the groups were significant. Differential metabolites were selected with a |log2FC| >0.58 and *P* < 0.05, the standard of False Discovery Rate (FDR) was less than 0.05. Pathway analysis of metabolomic data was conducted using MetaboAnalyst6.0 (http://www.metaboanalyst.ca/) to predict the enriched pathways of differential metabolites.

### 2.13 Metabolome, SCFAs, and intestinal microbiota correlation analysis

Correlative analysis between microbial species, SCFAs and metabolites was performed using Spearman’s correlation coefficient in R version 4.1.3 (corrplot: 0.92).

### 2.14 Statistical analysis

Statistical analysis was performed using GraphPad Prism 9 software, and results are presented as mean ± standard deviation. One-way ANOVA was used for multiple group comparisons, followed by the non-parametric Kruskal–Wallis test for post-hoc analysis. Intergroup comparisons were determined using Student’s t-test for significance. The FDR was employed to effectively manage the false positive rate arising from multiple hypothesis testing, with an FDR threshold set at <0.05 for significance. Additionally, based on the research objectives and practical needs, power analysis software was used to verify that a sample size of 12 mice per group would meet the expected effect size and variability power levels. Statistical significance is indicated as follows: *P < 0.05, **P < 0.01, ***P < 0.001 vs. the model group; ^#^ *P* < 0.05, ^##^ *P* < 0.01, ^###^ *P* < 0.001 vs. the control group.

## 3 Results

### 3.1 Chemical composition analysis of LSRZT

**FIGURE 1:**
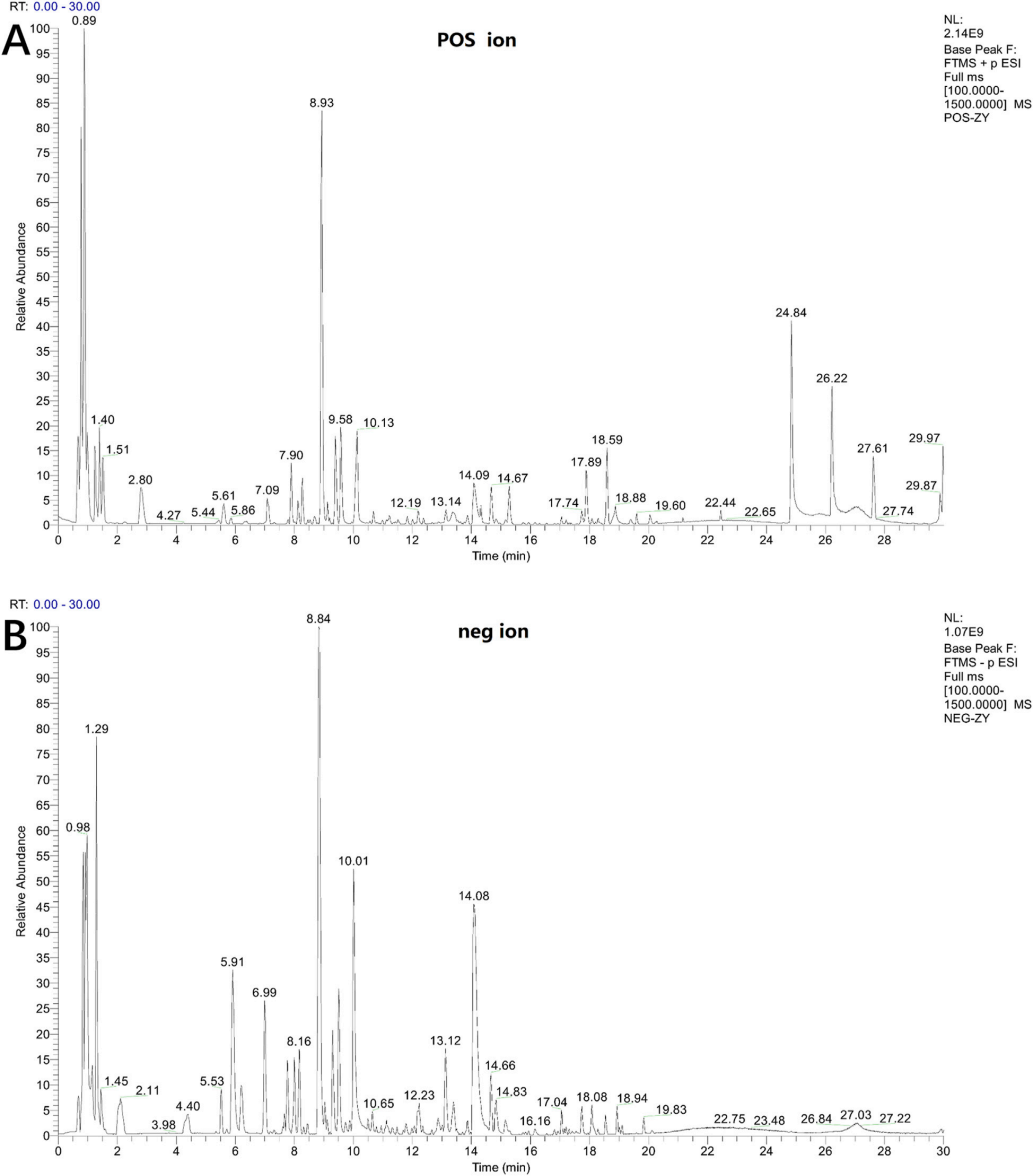
Base peak ion chromatogram of LSRZT detected in positive **(A)** and negative **(B)** mode.

The chemical composition of LSRZT was identified by UPLC-MS/MS analysis. Base Peak Ion of LSRZT in positive and negative ion modes were shown in Figures 1A,B, and 42 components of LSRZT were listed in Supplementary Table S1. 13 components were detected in negative ion mode, 13 components were detected in positive ion mode and 16 components were detected in positive and negative ion mixed mode. Flavonoids and Isoflavonoids, Organooxygen compounds, Prenol lipids was the main chemical component, accounting for 40.48%, 21.43%, 21.43% respectively.

### 3.2 Network pharmacology analysis

By searching from TCMSP, using BATMAN-TCM database to verify and remove false positives, 276 genes related to STD components were identified. In the Gene Cards database, “Sjögren’s Syndrome” was used as the key word to search, and after the string database was verified and the false positive was removed, a total of 1,062 disease genes related to SS were identified. The above genes were imported into Venn database, and the intersection of 76 common genes was obtained, as shown in Supplementary Figure S1A. Uploading the intersection gene to Cytoscape 3.7.2 software, and creating a protein interaction diagram, as shown in Supplementary Figure S1B. GO and KEGG enrichment analysis of intersection genes were carried out by DAVID data platform and R language, and the results were shown in Supplementary Figures S1C and S1D. GO analysis indicated that LSRZT primarily treated SS by modulating various factors, including cytokines, signal transduction receptor activators, receptor regulatory activities, and cytokine activities. It also influenced biological pathways such as phosphatase binding, protein phosphatase binding, chemokine receptor binding, and TNF receptor binding. KEGG analysis indicated that LSRZT mainly treated SS through TNF, IL-17, AGE-AGE receptor, HIF-1, and C-lectin receptor. The release of IL-17 and TNFSF13 is related to gut flora imbalance and intestinal mucosal immune dysfunction. Therefore, we further explored the mechanism of LSRZT in treating SS through gut flora.

### 3.3 LSRZT regulated immune function, reduced inflammation levels and alleviated damage

#### 3.3.1 Effects on daily water consumption and salivary flow rate in NOD/LtJ mice

**FIGURE 2:**
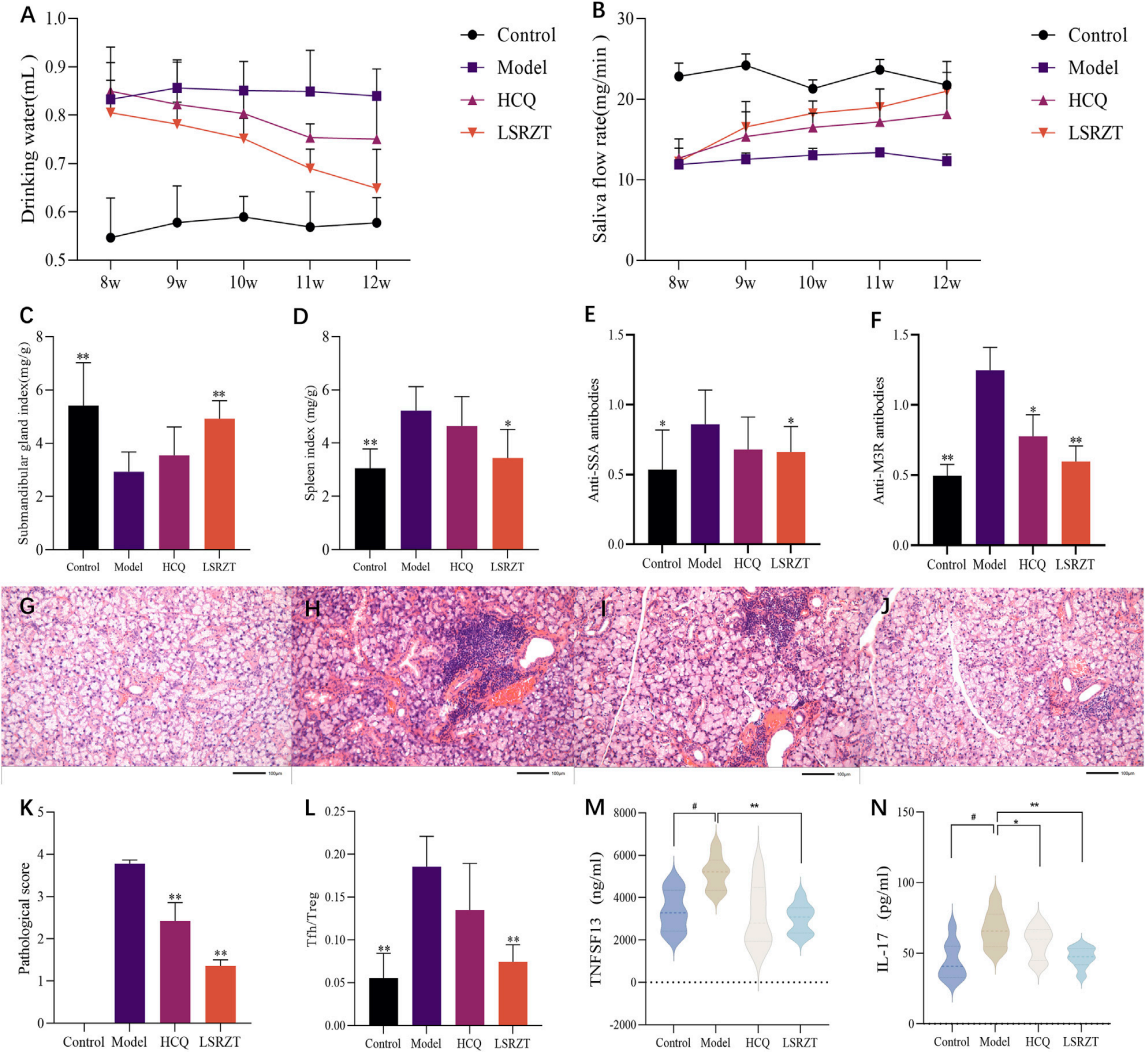
LSRZT regulates immune function, reduces inflammation levels and alleviates damage. **(A)** Changes in water intake in mice. **(B)** Changes in salivary flow rate in mice. **(C)** Submandibular gland index. **(D)** Spleen index. **(E,F)** The levels of anti-SSA antibodies **(E)** and anti-M3R antibodies **(F)** were determined by ELISA. **(G–J)** HE staining in the submandibular gland tissues. **(G)** The Control group. **(H)** The Model group. **(I)** The HCQ group. **(J)** The LSRZT group. **(K)** Submandibular gland histopathological score. **(L)** Tfh/Treg cell ratio. **(M,N)** The levels of inflammatory cytokines TNFSF13 **(M)** and IL-17 **(N)** were determined by ELISA. *P < 0.05, **P < 0.01, ***P < 0.001 vs. the Model group; ^#^ *P* < 0.05, ^##^ *P* < 0.01, ^###^ *P* < 0.001 vs. the Control group.

In the 8th week, compared to the Control group, the average daily water intake of mice in the Model, LSRZT, and HCQ groups significantly increased. From the 9th to the 11th week, the average daily water intake of mice in the LSRZT and HCQ groups gradually decreased, falling below that of the Model group. In the 12th week, the water intake of the LSRZT group was lower than that of the HCQ and Model groups, approaching that of the Control group. The results indicated that LSRZT can significantly alleviate dry mouth in NOD/LtJ mice (Figure 2A).

In the 8th week, there was no significant difference in salivary flow rates among the Model group, HCQ group, and LSRZT group, all of which were lower than the Control group. From the 9th to the 11th week, the salivary flow rates of the HCQ and LSRZT groups gradually increased, being higher than the Model group but lower than the Control group. In the 12th week, the salivary flow rate in the LSRZT group significantly increased, approaching that of the normal group and being higher than that of the HCQ and Model groups. The results indicated that LSRZT can significantly increase saliva secretion in NOD/LtJ mice (Figure 2B).

#### 3.3.2 The effect of LSRZT on the submandibular gland and spleen index in NOD/LtJ mice

As shown in Figures 2C,D, compared to the Control group, the submandibular gland index of the Model group was significantly reduced (*P* < 0.01), while the spleen index was significantly increased (*P* < 0.01). Compared to the Model group, the HCQ group showed an increase in submandibular gland index and a decrease in spleen index, with no statistically significant difference (*P* > 0.05). The LSRZT group had a significantly increased submandibular gland index compared to the Model group (*P* < 0.01), while the spleen index was significantly decreased (*P* < 0.01).

#### 3.3.3 LSRZT reduced the levels of anti-SSA and anti-M3R antibodies

Testing for the level of anti-SSA and anti-M3R antibodies by ELISA. The results (Figures 2E,F) showed that compared to the Control group, the Model group had elevated levels of anti-SSA and anti-M3R antibodies (anti-SSA: *P* < 0.05, anti-M3R: *P* < 0.01); compared to the Model group, the HCQ group showed a decreasing trend in anti-SSA and anti-M3R antibodies levels (anti-SSA: *P* > 0.05, anti-M3R: *P* < 0.05), while the LSRZT group had reduced levels of anti-SSA and anti-M3R antibodies (anti-SSA: *P* < 0.05, anti-M3R: *P* < 0.01), which were lower than the HCQ group.

#### 3.3.4 LSRZT relieved submandibular gland tissue damage

The results of HE staining (Figures 2G–J) showed that there was no lymphocyte infiltration in the submandibular glands in the Control group, and the cells were arranged in a regular and orderly manner. The number of lymphocyte infiltrates in the Model group was visible. The increase in area, the difference in the size of the acinar and the destruction of the structure. Compared with the Model group, there was no significant difference in the number and area of lymphocyte infiltrates in the HCQ group. Compared with the Model group, the number of lymphocyte infiltration foci in the LSRZT group was reduced, the area was reduced, and the structure was more intact. The pathological score (Figure 2K) showed that the pathological score of the submandibular gland tissue in the Model group was significantly higher than that of the Control group. Compared with the Model group, the pathological score of the submandibular gland in the LSRZT group was significantly lower than that of the Model group (*P* < 0.01). The HCQ group was also significantly lower than the Model group (*P* < 0.01), higher than the LSRZT group. The results showed that LSRZT can effectively improve the lymphocyte infiltration and protect the function of submandibular gland.

#### 3.3.5 LSRZT regulated the Tfh/Treg balance in NOD/LtJ mice

As shown in Figure 2L, compared to the Control group, the Model group exhibited a significantly higher proportion of splenic T follicular helper (Tfh) cells (CD3^+^CD4^+^CD279^+^CXCR5^+^) and a reduced frequency of regulatory T (Treg) cells (CD3^+^CD4^+^CD25^+^Foxp3^+^), resulting in a markedly elevated Tfh/Treg ratio (*P* < 0.01). Following therapeutic intervention, the HCQ group demonstrated a non-significant downward trend in Tfh/Treg ratios compared to the Model group (*P* > 0.05), whereas the LSRZT group showed a significant reduction in Tfh/Treg ratios (*P* < 0.01). The results indicated that LSRZT effectively modulates immune homeostasis by suppressing pathogenic Tfh cell expansion while enhancing Treg-mediated immunoregulation.

#### 3.3.6 LSRZT reduced the expression of inflammatory factors

The expression levels of TNFSF13 and IL-17 in the four groups were measured using ELISA. As shown in Figures 2M,N, compared to the Control group, the expression levels of TNFSF13 and IL-17 in the Model group were significantly increased (*P* < 0.05). Relative to the Model group, the expression levels of TNFSF13 and IL-17 in the HCQ group were reduced; the expression levels of TNFSF13 and IL-17 in the LSRZT group were significantly decreased (*P* < 0.01) and were lower than those in the HCQ group. The results suggested that LSRZT can significantly lower the expression levels of TNFSF13 and IL-17.

### 3.4 LSRZT regulated SCFAs in NOD/LtJ mice

**FIGURE 3:**
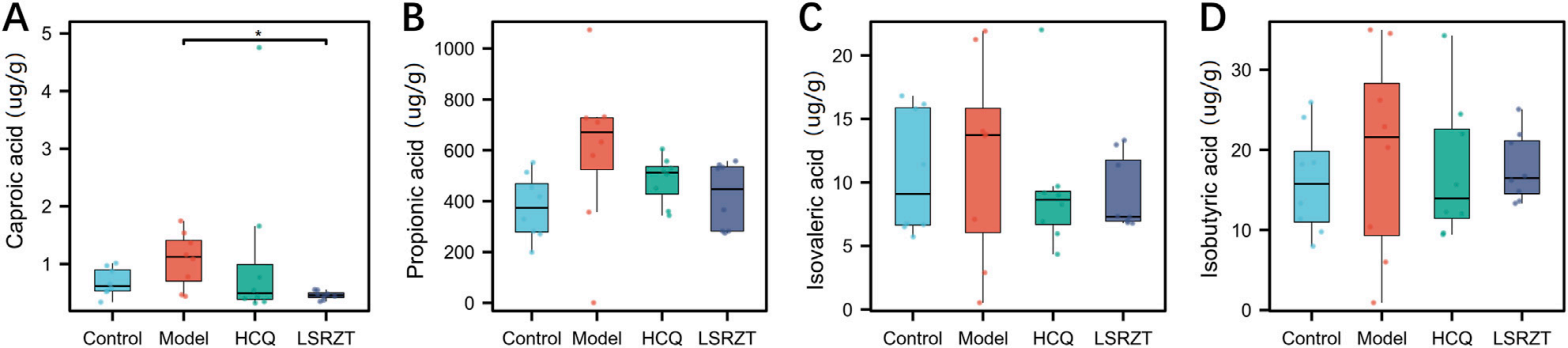
LSRZT regulated the levels of SCFAs in NOD/LtJ mice. **(A)** Caproic acid. **(B)** Propionic acid. **(C)** Isovaleric acid. **(D)** Isobutyric acid. *P < 0.05, **P < 0.01, ***P < 0.001 vs. the Model group; ^#^ *P* < 0.05, ^##^ *P* < 0.01, ^###^ *P* < 0.001 vs. the Control group.

SCFAs in the feces of NOD/LtJ mice were determined by GC-MS. As shown in Figures 3A–D, compared to the Control group, the Model group significantly increased the concentrations of propionic acid, isovaleric acid, isobutyric acid, and caproic acid. After LSRZT treatment, the contents of caproic acid in feces of NOD/LtJ mice was significantly reduced (*P* < 0.01), while The content of propionic acid, isovaleric acid, and isobutyric acid showed a non-significant decreasing difference compared to the Model group (*P* > 0.05). The results indicated that LSRZT can reduce the levels of SCFAs in NOD/LtJ mice, and caproic acid may be a potential therapeutic target.

### 3.5 LSRZT regulated intestinal dysbacteriosis in NOD/LtJ mice

**FIGURE 4:**
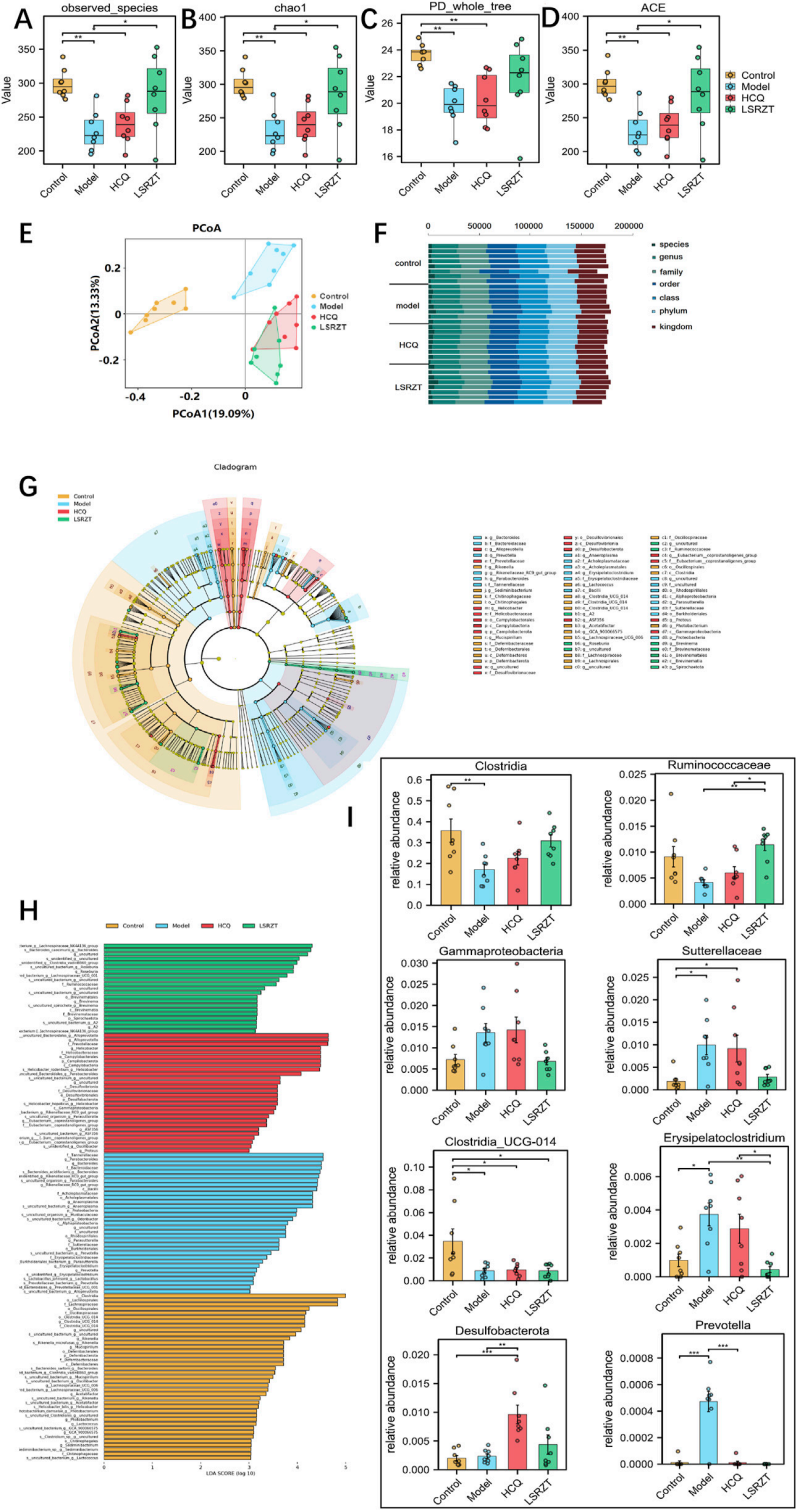
Assaying key gut microbiota associated with dysbiosis in NOD/LtJ mice regulated by LSRZT through 16S rRNA sequencing. **(A–D)** Alpha-Diversity analysis. **(A)** Observed_species index. **(B)** Chao1 index. **(C)** PD_whole_tree index. **(D)** ACE index. **(E)** Beta-Diversity analysis. **(F)** Proportions at the gut microbiota for each group. **(G)** LEfSe analysis that cladogram showing the phylogenetic distribution of microbiota associated with the four groups. **(H)** Histogram of the LDA scores. **(I)** Relative abundance of key bacteria at the genus, family, class, and phylum levels. *P < 0.05, **P < 0.01, ***P < 0.001 vs. the Model group; ^#^ *P* < 0.05, ^##^ *P* < 0.01, ^###^ *P* < 0.001 vs. the Control group.

In order to assess the regulatory effect of LSRZT on the intestinal microbiota, we first examined the alpha diversity of the gut flora in the all groups, compared to the Control group, the observed species index, chao1 index, PD_whole_tree index and ACE index was significantly lower in the Model group (*P* < 0.01), while it was significantly higher in the HCQ and LSRZT groups (observed species, chao1, and ACE index: *P* < 0.05; PD_whole_tree index of HCQ: *P* < 0.01), the LSRZT group is closer to the control group (Figures 4A–D). Beta diversity analysis was conducted to examine the similarities and differences in community composition among various groups. The results of the PCoA indicated that the gut flora of NOD/LtJ mice underwent significant changes. Following treatment, the gut flora shifted away from the Model group and moved closer to the Control group, suggesting that LSRZT and HCQ enhanced the diversity of gut flora in NOD/LtJ mice (Figure 4E). The microbial populations at the species, genus, family, order, class, phylum, and kingdom levels of the Control, Model, HCQ, and LSRZT groups were evenly distributed, there was an abundance of microbes sufficient to distinguish each individual (Figure 4F).

In order to find the key system types and biomarkers of gut flora in different groups, the high-dimensional categories were compared and analyzed by LEfSe to reveal variations in taxa abundance among the four groups, and found that there were significant differences in bacterial community dominance between groups (Figures 4G,H), detailed LEfSe results are provided in Supplementary Table S2. The results showed that Clostridia, Ruminococcaceae, Gammaproteobacteria, Sutterellaceae, Clostridia_UCG-014, Erysipelatoclostridium, Desulfobacterota, and Prevotella were the key gut microbiota, detailed ANOVA results were provided in Table 1.

**TABLE 1:** Comparison of the microbial of 8 significant difference bacteria in four groups by ANOVA.

Classification	Microbial name	P Value	FDR	Fisher’s LSD
class	Campylobacteria	0.000582	0.002851	HCQ - Control; LSRZT - Control; HCQ - Model; LSRZT - Model
	Clostridia	0.007076	0.021228	Control - HCQ; Control - Model; LSRZT - Model
phylum	Desulfobacterota	0.000352	0.002288	HCQ - Control; HCQ - LSRZT; HCQ - Model
genus	Prevotella	1.18E-09	4.00E-08	Model - Control; Model - HCQ; Model - LSRZT
	Erysipelatoclostridium	0.001179	0.010689	HCQ - Control; Model - Control; HCQ - LSRZT; Model - LSRZT
	Clostridia_UCG-014	0.006347	0.039238	Control - HCQ; Control - LSRZT; Control - Model
family	Ruminococcaceae	0.002356	0.016017	Control - Model; LSRZT - HCQ; LSRZT - Model
	Sutterellaceae	0.00709	0.030131	HCQ - Control; Model - Control; HCQ - LSRZT; Model - LSRZT

As shown in Figure 4I, the abundance of Sutterellaceae and Erysipelatoclostridium in NOD/LtJ mice increased significantly, but decreased significantly after administration of HCQ and LSRZT, especially LSRZT decreased to the level of healthy mice, especially, the abundance of Erysipelatoclostridium in the LSRZT group was lower than the Control group (*P* < 0.01). The Ruminococcaceae and Clostridia abundance of NOD/LtJ mice decreased significantly, but increased markedly after the administration of HCQ and LSRZT, LSRZT increased more significantly than HCQ, which was closer to healthy mice. Notably, the abundance of Ruminococcaceae in the LSRZT group was higher than the Control group (*P* < 0.01). The abundance of Gammaproteobacteria was significantly increased in NOD/LtJ mice. After administering LSRZT, the abundance significantly decreased and was closer to the Control group. In the abundance of Clostridia_UCG-014, Desulfobacterota and Prevotella, after administering LSRZT and HCQ, there were no significant changes.

### 3.6 LSRZT altered the metabolic profile of NOD/LtJ mice

**FIGURE 5:**
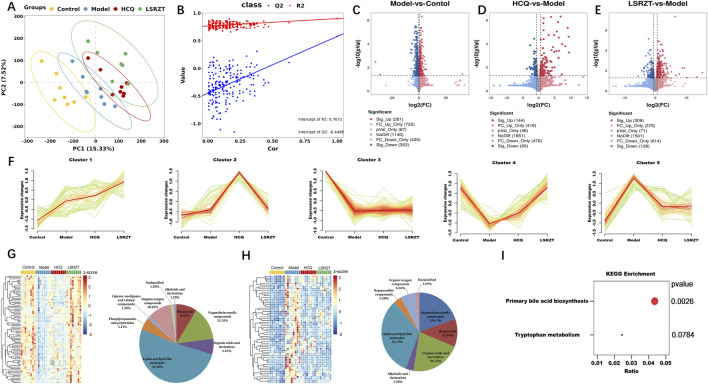
Assaying key metabolic pathways for improving metabolic abnormalities in NOD/LtJ mice through metabolomics. **(A)** PC analyses of fecal metabolites. **(B)** Class analyses of fecal metabolites. **(C–E)** Volcano plot comparing each group with the Model group. **(C)** Model vs. Control. **(D)** HCQ vs. Model. **(E)** LSRZT vs. Model. **(F)** Hierarchical clustering illustrating the distinct expression patterns of the four groups. **(G)** Heatmap and Pie chart of Cluster 4 that identified as the upregulated cluster. **(H)** Heatmap and Pie chart of Cluster 5 that identified as the downregulated cluster. **(I)** KEGG pathway enrichment scatter plot. *P < 0.05, **P < 0.01, ***P < 0.001 vs. the Model group; ^#^ *P* < 0.05, ^##^ *P* < 0.01, ^###^ *P* < 0.001 vs. the Control group.

A total of 32 fecal samples and 3 QC samples were analyzed using UHPLC-Q-TOF/MS. The score plot of the PLS-DA model was used to analyze the first two principal metabolites of the four groups, indicating effective separation of the principal metabolites (Figure 5A). PLS-DA analyses of fecal metabolites among the four groups were conducted (R2 = 0.901, Q2 = 0.578, Intercept R2 = 0.767, Intercept Q2 = −0.500), demonstrating significant differences in fecal metabolites across the four groups (Figure 5B). 2,998 metabolites in ESI+ and ESI- modes were identified, of which 447 differentially abundant metabolites (VIP >1, FDR <0.05) were subjected to statistical analysis. Volcano plots revealed distinct metabolic profiles across groups under stringent thresholds (|log2FC| >0.58, *P* < 0.05, and FDR <0.05). Compared to the Control group, 281 metabolites were upregulated and 353 metabolites were downregulated in the Model group (Figure 5C); compared to the Model group, 144 metabolites were upregulated and 65 metabolites were downregulated in the HCQ group (Figure 5D), 309 metabolites were upregulated and 128 metabolites were downregulated in the LSRZT group (Figure 5E).

To identify specific metabolites that distinguished the HCQ and LSRZT groups, mFuzz was used to cluster commonly identified metabolites from all four datasets into five significant discrete clusters with quantified values (Figure 5F). Cluster 4 was identified as the upregulated cluster when comparing LSRZT to the HCQ group. After combining the filter results and significantly altering the metabolites, 78 unique metabolites included lipids and lipid-like molecules (51.28%), organ heterocyclic metabolites (14.1%), organic oxygen metabolites (10.26%), benzenoids (8.97%), and other markers (Figure 5G). Cluster 5 was selected as the downregulation filter in the HCQ and LARZT groups. After combining the filter results and significantly altering the metabolites, 61 unique metabolites included lipids and lipid-like molecules (32.78%), organic acids and their derivatives (21.31%), organ heterocyclic metabolites (19.27%), benzenoids (11.48%), and other markers (Figure 5H). KEGG pathway enrichment analysis was performed on the differentially abundant metabolites, primary bile acid biosynthesis and tryptophan metabolism were key pathways for LSRZT treatment of SS, especially, primary bile acid biosynthesis have the strongest correlation (*P* < 0.01, impact >0.01) (Figure 5I).

### 3.7 Correlation analysis between the SCFAs, metabolites, and microbiota

**FIGURE 6:**
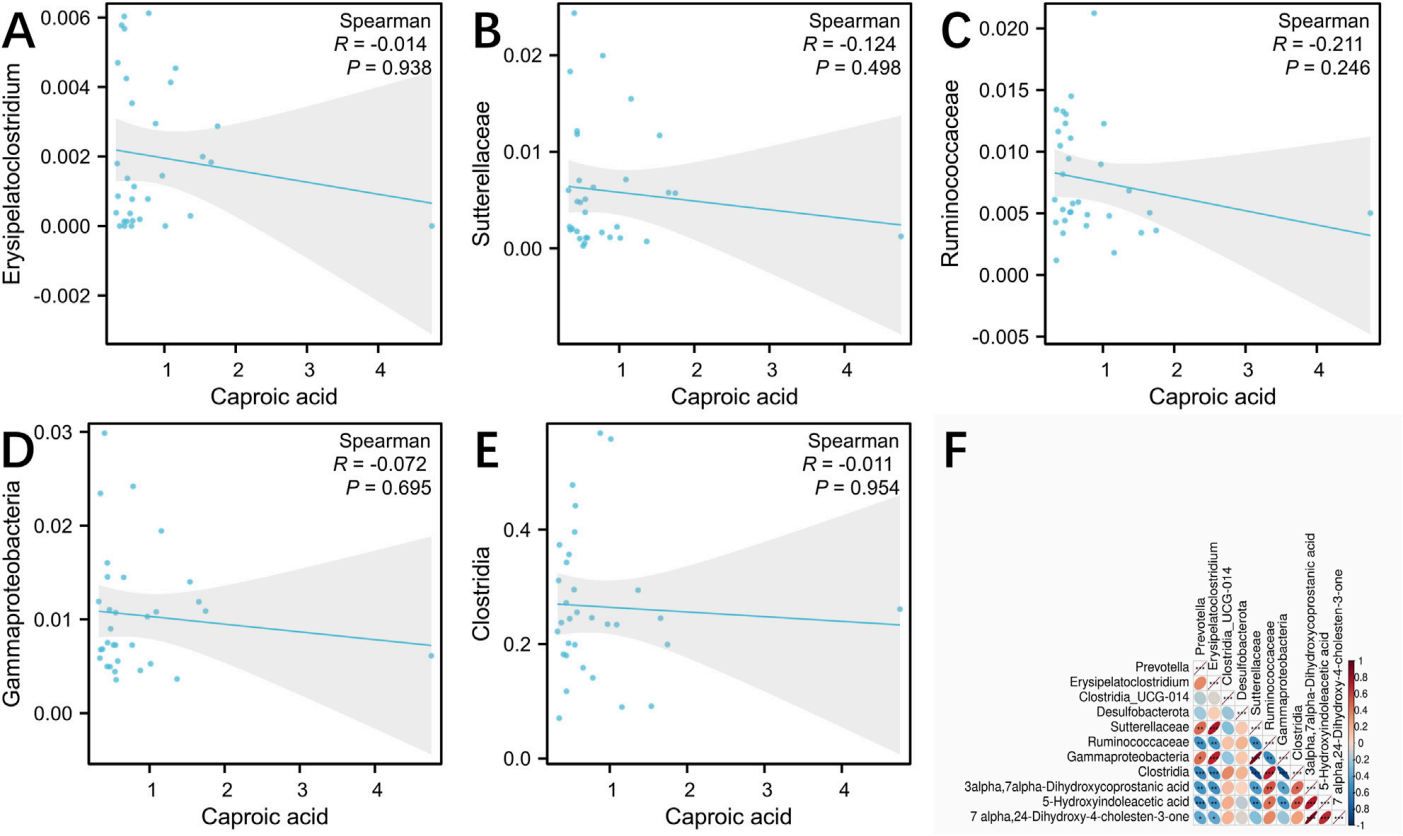
**(A–E)** Spearman correlation analysis between the gut microbiota and caproic acid. **(A)** Erysipelatoclostridium, **(B)** Sutterellaceae, **(C)** Ruminococcaceae, **(D)** Gammaproteobacteria, **(E)** Clostridia. **(F)** Spearman correlation analysis between the gut microbiota and primary bile acid biosynthesis, tryptophan metabolism. *P < 0.05, **P < 0.01, ***P < 0.001 vs. the Model group; ^#^ *P* < 0.05, ^##^ *P* < 0.01, ^###^ *P* < 0.001 vs. the Control group.

To investigate the relationship between SCFAs, metabolites and microbiota, we conducted the correlation analysis using Spearman correlation coefficients. Caproic acid may be the main target of LSRZT in the treatment of SS, and Spearman correlation analysis indicated that Clostridia, Ruminococcaceae, Sutterellaceae, Gammaproteobacteria, and Erysipelatoclostridium were negatively correlated with caproic acid (Figures 6A–E). However, there is no strong correlation between microbiota and caproic acid. In the primary bile acid biosynthesis pathway, the metabolites most closely associated with the significant microbiota were 3a, 7a-Dihydroxycoprostanic acid and 7a, 24-Dihydroxy-4-cholesten-3-one, whereas in the tryptophan metabolic pathway, it was 5-Hydroxyindoleacetic acid. The abundance of Clostridia and Ruminococcaceae was positively correlated with 3a, 7a-Dihydroxycoprostanic acid, whereas the abundance of Sutterellaceae, Gammaproteobacteria, and Erysipelatoclostridium was negatively correlated with 3a, 7a-Dihydroxycoprostanic acid, 7a, 24-Dihydroxy-4-cholesten-3-one and 5-Hydroxyindoleacetic acid (Figure 6F). The results showed that these microbiota fully participated in primary bile acid biosynthesis metabolism.

## 4 Discussion

In this study, we used SCFAs, 16S rRNA and metabonomics to explore the mechanism of LSRZT on SS, we found caproic acid was the main target, Erysipelatoclostridium and Ruminococcaceae were the important gut microbiota and primary bile acid biosynthesis metabolism was a main pathway for LSRZT to treat SS. Therefore, we thought that LSRZT reduces SS characteristics of NOD/LtJ mice by regulating the level of caproic acid and primary bile acid biosynthesis metabolism, and the abundance of Erysipelatoclostridium and Ruminococcaceae.

The amount of water intake, saliva flow rate, submandibular gland index, and spleen index are the main indicators for diagnosing SS. The study showed that LSRZT can reduce water intake in NOD/LtJ mice, increase saliva flow rate, and effectively alleviate dry mouth symptoms, LSRZT can improve the submandibular gland index and reduce the spleen index, and protect the functions of the submandibular gland and spleen. Anti-SSA is one of the standards for diagnosing SS, and the immunological characteristics of SS are primarily characterized by the presence of anti-SSA and anti-SSB antibodies, which manifest as hypergammaglobulinemia mainly due to elevated immunoglobulin G (Kramer et al., 2025). M3R is also an important parameter for assessing submandibular gland function, as the submandibular gland is a type of salivary gland, contributing about one-third of the total saliva volume, and M3 is the main receptor involved in regulating saliva secretion (Shaalan et al., 2018). This study found that under LSRZT intervention, the levels of anti-SSA and anti-M3R antibodies significantly decreased, confirming that LSRZT has the effect of restoring the levels of autoantibodies. Tfh cells are a subset of CD4^+^ helper T lymphocytes that play an important role in the severity of SS. Study found that the percentage of Tfh cells in the peripheral blood of SS patients increases with the severity of the disease (Fonseca et al., 2018). Treg cells are a special subset of T cells that perform negative regulatory functions; they inhibit autoimmune responses by secreting the inhibitory cytokine IL-10 and play a crucial role in maintaining immune tolerance and the negative regulation of immune responses, preventing the occurrence of autoimmune diseases (Romano et al., 2019). Tfh cells and Treg cells have opposing roles in promoting and inhibiting inflammatory responses, respectively. The imbalance between Tfh and Treg cells plays an important role in inflammatory diseases and may contribute to and promote the damage process of salivary gland acinar structures in SS patients (Li et al., 2021; Liu et al., 2008). The study indicated that LSRZT can downregulate the Tfh/Treg ratio, balance the homeostasis of Tfh/Treg, and delay glandular damage.

The occurrence and progression of SS are closely linked to inflammation, primarily characterized by lymphocytic infiltration in the exocrine glands. Research indicated that a significant number of T cells infiltrate submandibular glands, accompanied by the expression of various pro-inflammatory cytokines in SS mice (Iizuka et al., 2015). Further studies revealed elevated levels of Th17 cells, TNFSF13 and IL-17 in the salivary glands and peripheral blood of SS patients (Al-Megrin et al., 2020). The concentration of TNFSF13 and IL-17 in areas of lymphocytic infiltration are significantly increased (Ding et al., 2017). When the TNFSF13 and IL-17 gene are knocked out, mice exhibit no symptoms of SS or histopathological changes, and there is an increase in anti-inflammatory responses (Wang et al., 2009). TNFSF13 and IL-17 played a vital role in the pathogenesis of SS. In this study, the ELISA results indicated that LSRZT significantly lowered the levels of IL-17 and TNFSF13 in SS model mice and had effective anti-inflammatory properties and immune regulation. And the other results showed that LSRZT significantly improved the infiltration of lymphocytes in the submandibular gland, reduced inflammatory reaction, effectively delaying gland destruction and preserving salivary gland function.

Gut microbiota imbalance has been considered as one of the important pathogenic factors of SS. Meanwhile, inflammation plays an important role in regulating intestinal microbiota. A study demonstrated that the release of significant amounts of IL-17 and TNF acauses an imbalance in gut flora and disruptes the intestinal mucosal barrier (Singh et al., 2024). Sutterellaceae, Erysipelatoclostridium and Gammaproteobacteria belong to pro-inflammatory gut microbiota. Studies showed that Sutterellaceae can effectively attach to intestinal mucus and epithelial cells, stimulating these cells to release IL-17 and exhibiting a pro-inflammatory effect (Kaakoush, 2020; Hiippala et al., 2016). As a potential pathogen, Erysipelatoclostridium is positively correlated with TNF-a levels, Studies indicated that the abundance of Erysipelatoclostridium in diseased mice is significantly elevated (Cornick et al., 2019; Kaakoush, 2015). Gammaproteobacteria exhibit a relatively high tolerance to oxygen diffusing from the epithelial barrier, which can result in an ecological imbalance in the intestines and contribute to intestinal inflammation, the incidence of necrotizing enterocolitis was positively correlated with the abundance of Gammaproteobacteria (Liang et al., 2024; La Rosa et al., 2014). This was consistent with our study trend, LSRZT can effectively reduce the abundance of Sutterellaceae, Erysipelatoclostridium, and Gammaproteobacteria. Ruminococcaceae and Clostridia belong to anti-inflammatory gut microbes. Ruminococcaceae, as a member of Firmicutes, is one of the most abundant families in the order Clostridiales, which is associated with the maintenance of gut health (Biddle et al., 2013). Ruminococcaceae degrades complex carbohydrates, Both RuminococcaceaeUCG-002 and RuminococcaceaeUCG-014 produce butyrate, which plays a dominant role in colon health (Louis and Flint, 2009; G et al., 2018). Meanwhile, it can enhances mucus production and reduce the level of inflammation effectively (Crost et al., 2013). Clostridia is a butyrate-producing symbiont found in the human intestine. It has been safely utilized as a probiotic and exhibits significant anti-inflammatory effects, suggesting its potential to protect against or improve diseases (Stoeva et al., 2021). Study showed that the level of IL-1β and IL-6 decreased significantly following the intervention with Clostridia, the intervention regulated both the quantity and functional state of lamina propria dendritic cells in the intestinal mucosa of mice, thereby reducing the intestinal inflammatory response (Zhao et al., 2019). Gut microbiota c-Clostridia and f-Ruminococcaceae can protect the host from inflammatory damage due to SS driven hyperactivation of the dendritic cells, over secretion of the pro-inflammatory cytokines (IL-12, IFN-γ) and TLR activation (de Paiva et al., 2016). Our study showed that the levels of pro-inflammatory gut microbes increased and anti-inflammatory gut microbes decreased in model group, levels of pro-inflammatory gut microbes decreased and anti-inflammatory gut microbes increased after administration of HCQ and LSRZT, especially LSRZT was more obvious.

Caproic acid plays a significant role in inflammation and immune regulation, and has a close relationship with the gut microbiota. Caproic acid typically exhibits an anti-inflammatory effect, however, it can also induce a pro-inflammatory response, particularly through the stimulation of the MAPK, PI3K, or mTOR signaling pathways. These pathways are associated with the production of pro-inflammatory cytokines, such as IL-17, CXCL1, and CXCL2 (Li et al., 2018). These inflammatory factors are closely related to SS. IL-17 promotes the progression of SS by driving the proliferation of Th17 cells and inducing the expression of CXCL1/CXCL2 (Zhan et al., 2023). Therefore, the level of caproic acid may increase significantly in SS, and LSRZT improves the SS characteristics by reducing caproic acid levels. Study indicated that the level of caproic acid increased in infants with enterocolitis, following treatment, the level of caproic acid decreased, negatively correlated with the abundance of Bifidobacterium (Xiong et al., 2024). In a study involving patients with non-small cell lung cancer, the level of caproic acid decreased after immunotherapy, and Escherichia-Shigella was positively correlated with caproic acid (Ren et al., 2024). Bifidobacterium is negatively correlated with Gammaproteobacteria (Nagao-Kitamoto and Kamada, 2017), and Escherichia-Shigella belongs to the Gammaproteobacteria group. This is consistent with our research findings, which indicate that LSRZT can reduce the abundance of Gammaproteobacteria, and further suggests that LSRZT may lower caproic acid levels by regulating the abundance of Gammaproteobacteria.

Metabolites produced by gut microbiota are crucial molecular mediators between microbial communities and their hosts. Primary bile acid biosynthesis is an important pathway in bile acids (BAs) metabolism and serves as a crucial regulatory factor in maintaining intestinal function and modulating inflammatory responses (Li et al., 2023). Primary bile acid biosynthesis has antibacterial properties and can eliminate excess waste and toxic metabolites (Fuchs and Trauner, 2022). Deficiency in BAs can easily lead to mucosal immune disorders and induce inflammatory responses. Research showed that BAs can alter immune homeostasis by stimulating nuclear receptors Farnesoid X Receptor (FXR) and Takeda G Protein-Coupled Receptor 5 (TGR5), thereby reducing IL-17 levels (Sun et al., 2021). SS is characterized by autoimmune inflammation of the salivary and lacrimal glands, and abnormal FXR function may exacerbate glandular damage. TGR5 can inhibit inflammatory responses, and impaired Treg function in SS patients may be related to TGR5 dysregulation (Selmi and Gershwin, 2017). Therefore, BAs play an important role in the occurrence and development of SS. In this study, the metabolomics results indicated that LSRZT may treat SS by regulating the metabolic levels of primary bile acid biosynthesis, and the metabolic pathway is a key pathway for LSRZT to differentiate the treatment of SS from HCQ.

There is a strong relationship between gut flora and metabolites. BAs have a role in inhibiting bacterial growth can prevent bacteria from adhering to the top of the intestinal mucosa, and antimicrobial effects (Begley et al., 2005), and are important regulators of intestinal integrity, microflora and inflammation (Fuchs and Trauner, 2022). Research indicated that the abundance of Erysipelatoclostridium is negatively correlated with several BAs, and may contribute to the metabolic disorder of BAs (Li et al., 2023). Another study showed that a notable decrease in Ruminococcaceae has been associated with low BAs levels in the intestine (Bajaj et al., 2014). In the study, Ruminococcaceae was positively correlated with 3a, 7a-dihydrocoprostatic acid and 7a, 24-dihydroxy-4-cholesten-3-one, while Erysipelatoclostridium showed a negative correlation with these compounds. 3a, 7a-dihydrocoprostatic acid and 7a, 24-dihydroxy-4-cholesten-3-one were intermediate products of primary bile acid biosynthesis and play an important role in BAs metabolism (Mikov et al., 2006). Therefore, the study showed that LSRZT regulates primary bile acid biosynthesis by downregulating the abundance of Erysipelatoclostridium and upregulating the abundance of Ruminococcaceae to delay inflammatory reactions and reduce the severity of SS disease.

In summary, the study revealed the mechanisms of LSRZT in treating SS through multi-omics approaches such as 16s rRNA, SCFAs, and metabolomics. Although the study had limitations in sample size and collection methods, our study indicated that LSRZT has significant therapeutic potential in treating SS, providing new evidence for targeted TCM treatment of SS. Next, we will conduct in-depth mass spectrometry analysis of specific metabolites to identify the specific factors driving changes in these metabolites. We will also perform targeted interventions on specific metabolites to clarify their direct causal relationship in the treatment of SS with LSRZT.

## 5 Conclusion

Our study indicated that LSRZT can regulate immune function, reduce inflammatory responses, and protect the function of submandibular gland tissue. Furthermore, this study utilized 16S rRNA, SCFAs, and metabolomics to demonstrate that LSRZT can influence gut microbiota, regulate the levels of SCFAs and BAs, primarily by modulating the abundance of Erysipelatoclostridium and Ruminococcaceae, as well as levels of caproic acid and primary bile acid biosynthesis to treat SS. The study explored the mechanism of LSRZT in treating SS, which may open new avenues for TCM in treating SS and represents an important area for future research.

## Data Availability

The original contributions presented in the study are publicly available. This data can be found here: https://doi.org/10.6084/m9.figshare.29364809.v1.
