# Supplementary figures and images for: Mechanisms of Lushi Runzao decoction in Treating Sjögren’s syndrome by remodeling gut flora to regulate bile acid and short-chain fatty acid metabolism
Source: Front Pharmacol. 2025 Jun 26;16:1505642. doi: 10.3389/fphar.2025.1505642 (PMC12241085; doi:10.3389/fphar.2025.1505642)

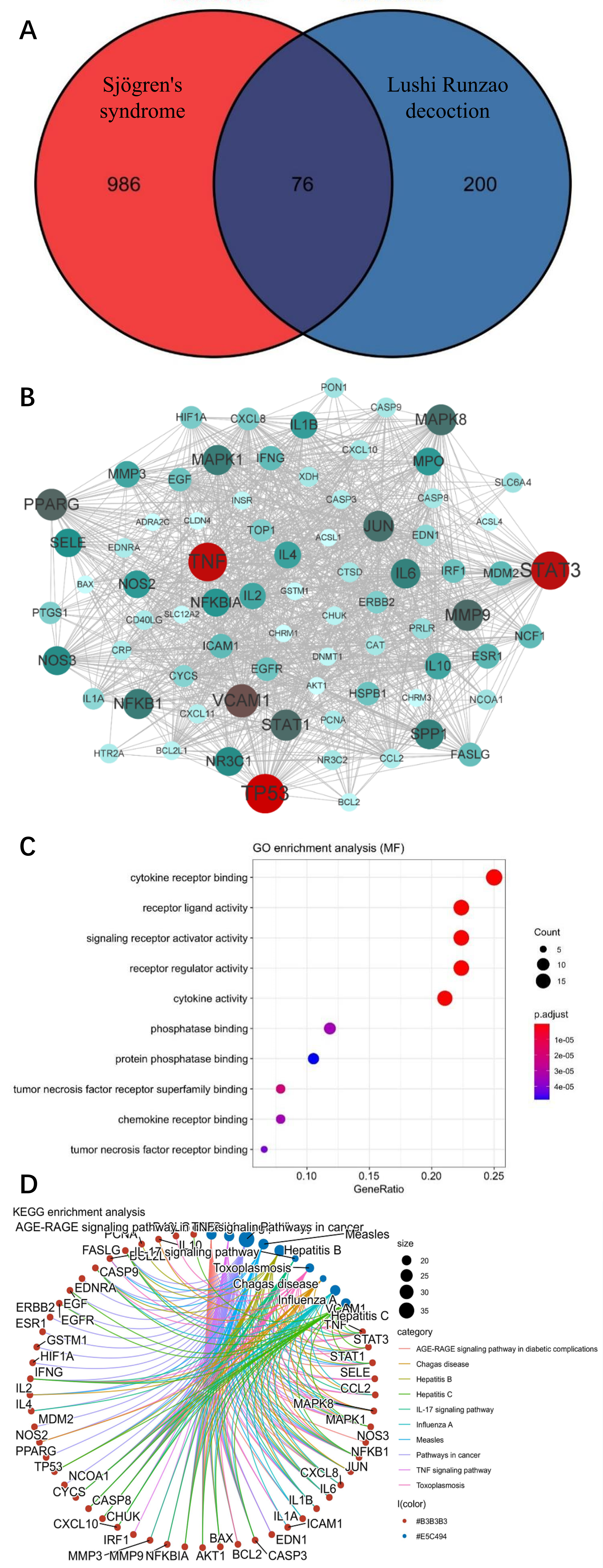

Supplement: Supplementary file 1 [file Supplementaryfile3.tif]
